# The social licence for data-intensive health research: towards co-creation, public value and trust

**DOI:** 10.1186/s12910-021-00677-5

**Published:** 2021-08-10

**Authors:** Sam H. A. Muller, Shona Kalkman, Ghislaine J. M. W. van Thiel, Menno Mostert, Johannes J. M. van Delden

**Affiliations:** Department of Medical Humanities, Julius Center for Health Sciences and Primary Care, University Medical Center Utrecht, Utrecht University, Universiteitsweg 100, 3584 CX Utrecht, The Netherlands

**Keywords:** Social licence, Data-intensive health research, Governance, Co-creation, Trust, Patient and public involvement

## Abstract

**Background:**

The rise of Big Data-driven health research challenges the assumed contribution of medical research to the public good, raising questions about whether the status of such research as a common good should be taken for granted, and how public trust can be preserved. Scandals arising out of sharing data during medical research have pointed out that going beyond the requirements of law may be necessary for sustaining trust in data-intensive health research. We propose building upon the use of a social licence for achieving such ethical governance.

**Main text:**

We performed a narrative review of the social licence as presented in the biomedical literature. We used a systematic search and selection process, followed by a critical conceptual analysis. The systematic search resulted in nine publications. Our conceptual analysis aims to clarify how societal permission can be granted to health research projects which rely upon the reuse and/or linkage of health data. These activities may be morally demanding. For these types of activities, a moral legitimation, beyond the limits of law, may need to be sought in order to preserve trust. Our analysis indicates that a social licence encourages us to recognise a broad range of stakeholder interests and perspectives in data-intensive health research. This is especially true for patients contributing data. Incorporating such a practice paves the way towards an ethical governance, based upon trust. Public engagement that involves patients from the start is called for to strengthen this social licence.

**Conclusions:**

There are several merits to using the concept of social licence as a guideline for ethical governance. Firstly, it fits the novel scale of data-related risks; secondly, it focuses attention on trustworthiness; and finally, it offers co-creation as a way forward. Greater trust can be achieved in the governance of data-intensive health research by highlighting strategic dialogue with both patients contributing the data, and the public in general. This should ultimately contribute to a more ethical practice of governance.

**Supplementary Information:**

The online version contains supplementary material available at 10.1186/s12910-021-00677-5.

## Background

The success of Big Data-driven or data-intensive health research relies greatly on public acceptance [1, 2]. This reliance has been highlighted by the public backlash following recent scandals in the field of data science. The Facebook-Cambridge Analytica data scandal in early 2018 lead to calls for more stringent regulation of tech companies’ use of data. In the area of health research, the case of *care.data* in the United Kingdom (UK) showed that mere compliance with the law is not enough for institutions to merit public trust when it comes to using citizens’ health data [2–4]. Increasingly, it appears to be warranted to go beyond regulations and law in order to internalise public norms [5].

To offer insight into the challenges faced by *care.data* medical sociologist Pam Carter and colleagues published a landmark paper in 2015 in which they introduce the concept of a so-called *social licence* to the domain of health data research. Carter and colleagues described this social licence as: ‘the expectations of society regarding the conduct and activities of corporations that go beyond the requirements of formal regulation’ [2]. They touched upon the concept’s necessary conditions, which include reciprocity, non-exploitation and service to the public good. This concept of social licence could be of great importance to data-intensive health research.

*Data-intensive* health research refers to the practice of large-scale capture, use, reuse, and/or linkage of a wide variety of health-related data on individuals. Putting the social licence into practice could help steer developments in data-intensive health research towards a more responsible and sustainable practice. The concept provides a highly warranted grounding for the development of *ethical governance*. However, firstly, what a social licence is, what work it does, and what its theoretical and practical challenges are, needs to be clarified. This can then inform how the social licence can be used in data-intensive health research.

## Main text

### Employing the social licence for data-intensive health research

In contrast to data-intensive health research itself, the concept of a social licence to operate in professional environments is not new. In the 1950s, American sociologist Everett Hughes used the term in his work on occupational relations [6, 7]. The concept was originally used to describe the level of approval from communities for certain professions to carry out activities that require more than regulatory permission. The social licence was later introduced into discussions about corporate social responsibility and sustainability [8]. The mining industry is probably the most notable example of a context in which the term has been widely used. Here it emerged that, in order to act responsibly, an additional *moral* duty exists—beyond compliance with laws and regulations. Only then could businesses gain ongoing approval for their activities from local stakeholders [9].

Over the years, the social licence has been discussed in depth in the context of commercial enterprises carrying out activities with an environmental risk [7, 8]. Now, following the problems encountered by *care.data* in the UK, Carter and colleagues have drawn the concept into the domain of health data research [2]*.* This *care.data* initiative to bundle up and share primary care medical records for research purposes faced criticism. Critics argued it disregarded patient objections, suffered from a low level of public awareness and transparency, and encountered problems in its communication and oversight strategies. The initiative was paused, and eventually abandoned entirely [10]. The *care.data* example underscores the need to recognise that public and patient trust is a serious prerequisite for realising successful health data research. Therefore, like the mining or logging industry, data-intensive health research must be viewed as an activity in which the public’s interest is at stake.

This is echoed in social contract theory, in which Big Data have recently been characterised as altering the nature of the implicit social contract underlying the relationship between governments and governed [11]. Whereas this social contract perspective focuses on the implications of Big Data on the politics of the state, the social licence approach is better suited to the public–private context of data-intensive health research. Nevertheless, by the same token, the social contract too stresses trust and justice in order to amend the adverse effects of Big Data on privacy and transparency [12].

Recent technological and analytical developments have rapidly increased the potential of data research to contribute to the common good. For example, the ease of data capture has increased with the transition from handwritten patient charts to electronic health records. Data from different sources can now, at least in theory, be combined to form large-scale databases covering the data of millions of patients around the world [13]. New analytical tools have become even more refined in order to identify associations and patterns within data. This has the potential to lead to new disease definitions, insights into disease aetiology, and relatively greater effectiveness of drug therapies [14, 15].

However, along with the potential benefits of data-intensive health research, potential risks arise from this type of research. Compared to the risks participants face in a clinical trial, the risks of data research are often less material in nature. People tend to be fairly supportive of sharing data for health research, even though they are also concerned about breaches of confidentiality and potential abuses of the data [16]. Studies consistently show that people want to be able to trust researchers to be transparent and responsible. People believe researchers to be capable of making an effort to provide information, of maximising data security, minimising risk, and holding accountable those who abuse and misuse the data [16]. With a focus on these values and concerns, a scrutiny of the concept of social licence forms an important step towards an ethical framework of governance.

### Methods

We performed a narrative review of the literature, with a systematic search and selection process, in order to explore the concept of social licence in data-intensive health research [17, 18]. This was followed by a critical analysis of the concept in order to identify the requirements to be able to use the concept for data-intensive health research.

#### Search and selection

On 15 February 2021, literature databases PubMed (MEDLINE), Embase, and Scopus were searched for publications describing the concept of social licence in health research. The term ‘social licence’, including its alternative spelling ‘social license’, was used to search titles, abstracts, and keywords of indexed publications (see Additional file 1). All references were imported into RefWorks and checked for duplicates. The search resulted in 220 unique publications. We used Rayyan for the selection of relevant articles (see Additional file 2). After selection, eight publications remained (see Additional file 3). One publication was added from other sources, resulting in a total of nine publications for the final analysis.

#### Data extraction and critical analysis

The analysis consisted of extracting the following information from the publications: (1) the definitions and components of a social licence; (2) the work a social licence allegedly needs to do; (3) challenges to achieving a social licence; and (4) what a social licence requires from data researchers. A constructive critical analysis was performed based upon these findings. We then formulated the next steps to operationalise the social licence in data-intensive health research.

### The definitions and components of a social licence

Our systematic literature search suggests little has been published in the biomedical scientific literature about the concept of a social licence. We structured our analysis around the nine publications that met our eligibility criteria. From the definitions provided by them, we arrived at the essential components of how a social licence is described (see Table 1).

**Table 1:** Definitions or descriptions of a social licence as provided in the biomedical literature

Authors	Year	Definition or description of social licence
Dixon-Woods and Ashcroft [19]	2008	Licence is granted to certain occupational groups to carry out particular activities. Social licence permits deviation from common modes of behaviour and is used by professions to claim a broad legal, moral and intellectual mandate: claims to define proper conduct in relation to matters concerned with their work.
Carter et al. [2]	2015	Licence is granted to certain occupational groups to carry out particular activities (according to Everett Hughes). Corporate social responsibility describes the concept of the ‘social licence to operate’ as the expectations of society regarding the conduct and activities of corporations that go beyond the requirements of formal regulation.
Ford et al. [20]	2019	Social licence theory proposes that the public expect that, in some circumstances, the conduct of groups or organisations should go further than the requirements of formal regulation, towards voluntary adherence to social codes of trustworthy and responsible behaviour. Where the public are satisfied that the motivations of the organisation are trustworthy, they grant a ‘social licence’ to operate.
Allen et al. [21]	2019	A privilege granted to an occupation or profession to do things other members of society are not allowed to do and which may not be morally acceptable in the wider society.
Krahe et al. [22]	2019	The extent to which entities (public and private) are constrained to meet societal expectations and avoid activities that societies deem unacceptable.
Paprica et al. [23]	2019	A social licence to operate is an informal agreement that is granted by communities and relevant stakeholders to an organisation to do certain work.
Xafis et al. [24]	2019	Social licence relates to the positive public expectations associated with the perceived legitimacy of activities that have broad societal impacts, and it also relates closely to trust, which, in turn, is enhanced via open, transparent communication.
Ballantyne and Stewart [25]	2019	Social licence permits some measure of flexibility in relation to common or expected modes of behaviour regarding data use. It describes whether a given data use is accepted by stakeholders.
Shaw et al. [26]	2020	Social licence refers to the informal permissions granted to institutions such as governments or corporations by members of the public to carry out a particular set of activities. Expectations thereby demand actions that go beyond existing legal rules to demonstrate concern for the interests of publics.

First of all, a social licence is characterised in the same way as any other kind of licence in the sense that it is *granted* by one party to another. To contrast the social licence with a legal licence that could come in the form of a physical object that can be bought, some describe the social licence more as a ‘privilege’ [21], a ‘permission’ or even an ‘informal agreement’ [23]. Three definitions specify the party granting the licence, namely, the ‘publics’ [26] or ‘communities and relevant stakeholders’ [23, 25]. All definitions name the party who is granted the social licence. Whereas some definitions simply refer to the parties receiving the social licence as ‘entities’ or ‘organisations’ [20, 23], as well as industries [25], others clarify the nature of these parties as occupational or professional [2, 19, 21]. One definition refers to ‘corporations’ specifically [2]. Two explicitly state that parties may be private but also public [22, 25].

The licence is granted to the receiving party to carry out ‘particular activities’ [2, 19]. No definition actually elucidates the activities any further than ‘to do things’ [21] or ‘doing certain work’ [23]. Yet some do qualify the nature of those ‘things’ a bit further. The activities for which a social licence is granted typically deviate from common modes of behaviour [19]. They constitute activities that have broad societal impacts [24], that other members of society are not allowed to do, or that may not be morally acceptable in the wider society [21, 22]. It is exactly because these activities are morally questionable or demanding that, in order for parties to act responsibly, they need to submit themselves to codes of conduct that go beyond legal or formal regulation [2, 20]. Four definitions mention the reasons why a social licence is granted or how a party can obtain a social licence. The party wishing to obtain a social licence for its activities will need to attest that those activities are acceptable in the eyes of society [22]. For activities to be deemed acceptable by society, they need to meet societal or public expectations [2, 22, 24, 25]. Three definitions explicitly mention that the public must be ‘satisfied’ that the activities to be carried out are ‘trustworthy’ [20, 25, 26]. One suggests special attention should be given to mistrust by particular communities [26]. Transparency, open communication [24, 26], engagement [24, 25], and data governance strategies are all emphasised as examples of how to gain trustworthiness [26]. A social licence is gained by trust in the voluntary adherence to social codes of trustworthy and responsible behaviour, reflecting ethical and societal standards [20]. After filling in each of the different components, we achieved a specification of a social licence for data-intensive health research, which reads:

A social licence in the context of data-intensive health research refers to the non-tangible societal permission or approval that is granted to either public or private researchers and research organisations. This allows them to collect, use, and share health data for the purpose of health research by virtue of those activities being trustworthy, by which is meant trusted to be in line with the values and expectations of the data subject communities, stakeholders, and the public.

### What work does a social licence do?

In the sparse literature on the subject, a social licence is described as serving the ambitious goal of ensuring data-intensive health research that is beneficial, ethical, responsible and sustainable [23]. In this account, however, a social licence is a moral requirement and not optional. By adding a focus on engagement and participation, giving greater attention to societal values and expectations, it thus becomes a norm in itself, beyond formal regulation [5, 27, 28]. As such, a social licence helps to establish the trustworthiness of the research project at large on top of the obligatory ethical safeguards. In doing so it involves all institutions and researchers of the research project in question [1, 29, 30]. By building trust in voluntary adherence to codes of conduct, there is less need to formalise good behaviour by introducing even more rules and regulations. A social licence can actually be viewed as allowing for more freedoms and to alleviate restrictions. Researchers, for instance, can assume they have a social licence to reuse data because of a positive public attitude resulting from their trustworthy and responsible behaviour.

More generally speaking, a social licence may also allow for a rebalancing of professional power within this relatively new area of health research [31]. For, to date, predominantly medical and scientific stakeholders have been in the position to determine the ethical boundaries of medicine, care and medical research. This division of moral labour has been justified, while at the same time, ‘lay’ and societal stakeholders have largely been left out [6, 32]. The purpose and requirements of a social licence therefore reside beyond the specificities of a particular law. In this way, a social licence fulfils a communicative, informational, and educational need towards both the medical practitioners and the public [33, 34]. In addition, it can work the other way too, opening up similar learning possibilities for the researchers and institutions involved in data-intensive health research. The social licence thus engenders mutually beneficial interaction and dialogue.

Attention to its social licence, therefore, can benefit data-intensive health research by fostering a greater supporting base, and alleviating the demanding framework within which researchers are restricted to work. However, the requirements for a social licence based on the literature remain vague. Elaborating on these requirements offers a direction towards establishing a framework for ethical governance predicated upon the social licence.

### Understanding the challenges to achieving a social licence

The case of *care.data* has highlighted several problematic issues involved in establishing a balance between formal, legal and administrative approaches to data-intensive governance frameworks, and the implications of a social licence. First of all, social licence warns us that current frameworks of governance risk losing their patients’ support. In an approach based upon social licence, the formulation of the role of citizens and patients in data research, the taking into account of their attitudes towards the ‘public good’, and their participation within data-intensive research is, instead, carried out from the bottom-up. As yet, these efforts are highly circumscribed by the perspective of the dominant, predetermined, data-intensive research goals [35, 36]. In times of rising costs and cutting back services this conforms to a general trend within the governance of health. Governance is becoming infused with norms instigating, or implicitly ‘nudging’, citizens to behave according to a predetermined, instrumentalist view of the public interest. Within this view, bottom-up input is merely seen as a useful tool for influencing behaviour. And so public interest is not seen as an end in itself, but rather as a means towards the end of making more data-intensive health research possible [37].

The contribution of patients to the expansion of data-intensive health research as *data subjects* similarly signifies a move away from well-established patient rights and ethical safeguards, and into uncharted territory [30]. For, in doing so, this move supplants a more ‘nuanced and delicate understanding of societal support for, and co-operation with, health research’ [2]. We should be especially cautious of this ethically challenging situation. At the same time as being encouraged to contribute their data to research, patients are approached as being responsible to do so [2]. This is likely to hinder the constructive engagement of those affected in situations where a social licence is deemed a worthy and valuable component of ethical governance [38].

Furthermore, misconceptions about patients may arise in discourse on policy such as that which was implicitly referred to in *care.data*’s National Health Service citizenship [2]. These misconceptions are found to be based on generalised assumptions about consent, support, and participation, originating in specific, particular, and limited studies [2, 39]. When transferred, and employed in practice, to legitimise data collection in the different context of data-intensive health research, this reaps uncertainty about the assumption of data-intensive health research’s public value for society. In addition, its status as a common good is bound to be problematic due to a greater multiplicity of goals. Some of these goals, especially those shaped and influenced by private interests, transcend the argument of pure public service. Also, considering, on the one hand, the data contributions of patients to research, and, on the other hand, contributions of data research to the public good, the risks and benefits involved in data sharing and access are perceived as unevenly distributed [2]. This is an especially pressing obstacle because it will result in sentiments of unfairness, and a lack of reciprocity [2]. As a consequence, trust is damaged. Moreover, the extent to which the protection of risks is able to mitigate these worrying developments, and with it the legitimacy of data-intensive health research, is questioned [40, 41].

Conversely, a social licence attributes special weight to the elements of public value and trust. Social licence sheds light on the caution as well as the nuanced and delicate appreciation needed for societal support and co-operation [19]. The starting point is that research must be seen once more as a subcategory of medicine, one which is particularly subject to more than regular medical risks alone. To be more specific, the context of data-intensive research adds renewed weight to, and intensifies, the risks to society and the communities which are involved [19, 42]. For not only do some health data research projects today involve co-operations which cross the public/private divide, they are also unprecedented in their international scale and usefulness [43]. As a new vein of research, this challenges the self-evidence that health research is in line with medicine’s entrenched orientation towards the collective [44]. These facets, therefore, have a significant effect upon the extent to which the institutional organisation and structure of data-intensive health research is up to its task of realising a research practice that is perceived to be ethically sound. The question is not whether prevailing ethical safeguards are fit for purpose, but rather to what extent they can adequately respond to the heightened social dimension of what ethical research today consists of.

Moreover, it is important to recognise the implications of the contemporary social structure for the role of medicine in handling the risks engendered by Big Data technology [13, 14, 45]. The relationship between patients’ and public attitudes and medical research has become far more critical and dynamic than was ever the case [46]. As a consequence, societal expectations cannot be assumed; they are less self-evidently supportive than before [46]. The value of the social licence lies in its response to this context. It highlights the fact that governance for data-intensive health research projects requires a revised and renewed mandate of its own [6]. This mandate goes beyond the one conferred upon them by law, and beyond a mandate assumed from prior health research. This renewal is needed in order to establish, or rather retain social legitimacy for medical research [19, 47]. Just as ‘lay’ evaluations of data-intensive medical practice rely greatly on the regulation and prevention of risks, such a mandate also contains a fragile mix of safety concerns and conceptions of the value of the research as a social good [40]. In this context, risk is ‘intricately and inevitably tied to the development of new organisational forms and logics that underlie the way that services are organised, managed and held accountable’ [48].

Yet the governance of health data is vulnerable to an implicit reiteration and reification—the changing of something abstract into something real—of a dominant medical rationality, principally by medical stakeholders [49, 50]. This endangers its social licence, since it means that data-intensive health research cannot be assumed to coincide with public value or the common good [31, 51]. Instead, for the governance of data-intensive health research, the presumed moral guidance and trustworthiness need to be validated, since the medical profession is just one stakeholder among many others [52]. The whole concept of a social licence centres upon the integration of stakeholder perceptions and evaluations—most essentially those of patients—on which a mandate can be built [9, 20]. This, in turn, rests on the recognition of an, albeit more fragile, ‘reflexively organized dialectic of trust and doubt, certainty and uncertainty’ [46]. Recognition of this dynamic is pivotal to the relationship between data-intensive health research and societal support—and, therefore, its confirmation as a public good.

Social licence, therefore, provides opportunities for an alignment between patients, non-medical and societal stakeholders, the public, and medical stakeholders. This is extremely important for sustaining an ethical practice of data-intensive health research.

### What a social licence requires from data researchers

What a social licence requires from the professionals involved, most importantly data researchers, ranges from abstract principles and public values [23], to more concrete and practical obligations. Such core values mentioned include reciprocity, non-exploitation and service to the public good [2], as well as transparency and inclusivity [20, 26]. Quintessentially, the literature confirms that social licence requires apt medical research regulation and governance in order to ensure data researchers adhere to these values [19]. Therefore, the use of novel and secure data management technologies is highlighted as key to achieving social licence. These practical mechanisms foster transparency and accountability [20]. Nevertheless, it must be recognised that public and patient trust and expectations do not exist in isolation. They do not belong just to one domain, institution, or project. Instead of being confined, trust and expectations are fluid, making it all the more important to direct greater effort to them altogether.

Going beyond regulation, the need to mitigate not only actual risk but also perceived risk, and the building of relations of trust are emphasised in the literature [22]. Recognising the importance of public engagement provides a first step to greater awareness of societal expectations and concerns [23, 53, 54]. On the patient level, more effort should be directed to identifying, and putting into context, the factors that play a key role in a person’s decision to share their personal health information for research [22, 34]. This includes recognising that trust is placed both in research organisations’ competence with the handling of data, as well as their motivations for data analysis [55]. Here, there could be an important role for data custodians meriting a social licence, in addition to other stakeholders [21].

In order for patient and public involvement with data governance and decision-making to play a meaningful role, close alignment is needed with stakeholder priorities and needs, at all stages of the research project. This is true for both the technological and governance dimensions of the research, as these are still often dominated by experts [20, 54]. This orientation fosters transparency by strengthening participation and accountability, two ingredients recognised as pivotal for building and maintaining trust [1, 23]. However, achieving trust requires far more than effort and awareness on the part of the key group of data researchers alone. Outside scrutiny of how the current handling of risks within the established structures and institutions of data governance can incorporate patient and public concerns is also required [4, 56].

### Next steps towards operationalisation

Social licence necessitates coping with the novel institutional risk of data-intensive health research. This means being able to address and pre-empt the perceived risks to society in order to establish sustainable regimes of governance [57]. However, a greater understanding of the role of the social licence, thus securing trust in relation to risks, takes on an essential position when putting the concept into practice [2, 19].

The literature has highlighted that trust forms an essential moral mechanism intrinsic to any ethical governance of data-intensive health research [1, 26]. Practically, trust is pivotal in data-intensive health research, as actions cannot be fully overseen nor governed. This is simply because it is not yet known, nor may ever fully be known beforehand, what actions will be concerned with [2, 45]. Regarding such high, uncertain, and unforeseeable risks, it has been pointed out that trust resides beyond abstract systems [58]. This is pivotal in our thinking about the fostering of trust in governance. Mostly, the contribution of research ethics in relation to governance boils down to the analysis of the values it promotes, leading to recommendations at a formal, structural and organisational level. Instead, social licence demands a thorough development of the concept of trust, shifting our attention towards an ethical governance which revolves around interpersonal vulnerability, moral agency, and voluntary reciprocity [58]. It requires a shift towards a softer side of organising ethical governance. This would encompass notions such as shared [5], or rather, reflexive health governance [59], which focuses on mutual learning, understanding of perspectives and actions, and lastly, communication, interaction, and deliberation. These notions are well suited to encapsulate this focus into a practical governance framework [59, 60].

This, firstly, necessitates a departure from the dominant focus on a complete confidence in systems, which weakens the prominence of trust as a moral imperative [61]. What is known as the architecture of governance, its formal structure, serves a lesser role in coping with doubt and uncertainty concerning data-intensive health research, than the perceptions of organisations and individuals. These are more crucial than is taken for granted [41, 62]. The legitimacy and accountability of data-intensive health research rely on its socially affirmed status as a worthy enterprise executed in the service of the public good [63]. This way forward stresses taking seriously the expectations concerning the conduct and regulation of research, institutional trust, and the assertation of public value in general, and incorporating these via effective means of governance [41].

Secondly, this orientation based on trust ‘makes sense only if that entity first develops trustworthiness and only if our trust in it is a result of that trustworthiness’ [29]. Here too, formal safeguards and concise, yet complex organisational structure, are less important than is often asserted [1, 30, 61]. They may, in fact, be counterproductive, doing even more harm than good in establishing conditions for trust to flourish [58]. Alternatively, a focus on trustworthiness flips the perspective, in which social licence implies a *performative* function for the ‘lay’ stakeholder. In this way patient involvement in governance facilitates *co-creating* what is considered as trustworthy [5, 64]. Further research is warranted into why and how stakeholders perceive, evaluate and act upon data-intensive health research as they do. Specifically, this type of research should qualify the relationship between trust and trustworthiness from a public and patient perspective.

Thirdly, stakeholder involvement *beyond* mere attention and conversation forms another important step towards greater integration of trust and trustworthiness [4, 45, 65]. The actual co-creation of data governance, based on strategic dialogue and discussion, may yield great promise in this direction. One particularly fruitful way in which this can be achieved is by organising what is known as ‘extended fora’ [45]. Such fora, in the true sense of a public meeting place, may include a widening array of stakeholders that are active within data-intensive health research. A multifaceted approach to the participation of patients and members of the public, involving individuals and communities [45], is promoted as vital for a socially responsible research process. It will, at the same time, prevent tokenism and unrepresentative participation [45, 54]. A reflective approach to these extended fora underscores the point that engagement forms a tool for meaningful, transparent consideration of public values, interests and concerns [54, 59]. This, in turn, facilitates a more genuine open exchange of information and greater equity in the relationship between science and public [1]. By pursuing shared decision-making, ways of working can be built upon the articulated requirements for a social licence. This could allow for extended public spaces to reflect upon issues, acting as a balance to professional power and enhancing trust.

Finally, it should be stressed that the social licence should not be regarded as something issued as a one-off, static permit for all future activities. Instead, it requires continuous maintenance. It is important to acknowledge that this is not a simple process. A social licence in one domain may or may not influence those in other domains. Nevertheless, it does provide space for much needed provisional fixed points for data governance. By ascertaining expectations, it can lead us towards shared reflective equilibria [66], importing greater trust, and, therefore, certainty of support for data-intensive health research.

## Conclusions

For a conceptual examination of social licence in the context of data-intensive health research, we explored the degree to which it relates to formulating norms, as well as its theoretical and practical implications for this field. Social licence grants moral liberties to researchers based on society’s trust in their activities. Those liberties in turn demand trustworthiness, coming with duties to act in ethically acceptable ways. This is defined as behaviour that addresses concerns about data privacy and risk, and that promotes public values such as transparency, reciprocity, inclusivity, and the common good. Furthermore, technical requirements are important in addressing data security concerns, because these affect perceived, as well as actual risk. However, these alone will not be sufficient to establish and maintain a social licence. Public engagement and co-creation of norms should also be heavily prioritised as duties in order to understand what expectations stakeholders and society at large hold. In this way, the joint action is possible which is needed in order to achieve public engagement and co-creation. The social licence cannot be taken for granted; it calls for action.

Putting these elements into practice can start with actively integrating stakeholders into all the stages of data governance. Yet this effort must be infused by the open exchange of information and perceptions, fostering trust, mutual learning, and enhancing communication in the relationship between science and the public. In this regard, readily understood information, that is of practical use, which matters, and is accessible to members of the public is important for the ability to hold researchers to a code of conduct drawn up by both parties. Besides the process, and the design of its implementation, greater attention should be given to aspects of interaction and dialogue occurring within specific contexts of a social licence [9]. The concept of social licence highlights just how important and fragile the linking of all these elements is. Achieving a sustainable ethical governance requires all involved to acknowledge these dimensions in order to be able to tackle them together.

## Supplementary Information

**Additional file 1:Table**: Table indicating search strings for databases used (15 February 2021).

**Additional file 2:Table:** Table detailing inclusion and exclusion criteria.

**Additional file 3:Figure:** Figure containing flow diagram of the selection of publications.

## Data Availability

Not applicable.

## Abbreviation

UK: United Kingdom
