# Supplementary material for: The social licence for data-intensive health research: towards co-creation, public value and trust
Source: BMC Med Ethics. 2021 Aug 10;22:110. doi: 10.1186/s12910-021-00677-5 (PMC8353823; doi:10.1186/s12910-021-00677-5)
Supplement: Supplementary file 1 — Additional file 1:Table: Table indicating search strings for databases used (15 February 2021). [file 12910_2021_677_MOESM1_ESM.docx]

Additional file 1. Table indicating search strings for databases used (15 February 2021).

| **Database** | **Search string** | **# Hits** |
| --- | --- | --- |
| PubMed | ("social license"[Title/Abstract]) OR ("social licence"[Title/Abstract]) | 60 |
| Embase | 'social license':ti,ab,kw OR 'social licence':ti,ab,kw | 49 |
| Scopus* | TITLE-ABS-KEY ( "social license" ) OR TITLE-ABS-KEY ( "social licence" ) AND ( EXCLUDE ( SUBJAREA , "ENVI" ) OR EXCLUDE ( SUBJAREA , "EART" ) OR EXCLUDE ( SUBJAREA , "ENER" ) OR EXCLUDE ( SUBJAREA , "BUSI" ) OR EXCLUDE ( SUBJAREA , "ENGI" ) OR EXCLUDE ( SUBJAREA , "ECON" ) OR EXCLUDE ( SUBJAREA , "AGRI" ) ) | 111 |
| *As Scopus is not a biomedical literature database and the results would otherwise become unmanageable, we limited our search by applying the following filters: exclusion of literature in the fields of environmental sciences, earth and energy, business, economics, engineering and agricultural sciences. | | |
