# Supplementary material for: The social licence for data-intensive health research: towards co-creation, public value and trust
Source: BMC Med Ethics. 2021 Aug 10;22:110. doi: 10.1186/s12910-021-00677-5 (PMC8353823; doi:10.1186/s12910-021-00677-5)
Supplement: Supplementary file 2 — Additional file 2:Table: Table detailing inclusion and exclusion criteria. [file 12910_2021_677_MOESM2_ESM.docx]

Additional file 2. Table detailing inclusion and exclusion criteria.

| **Inclusion criteria** | **Exclusion criteria** |
| --- | --- |
| Publication provides description of social licence in health data research | Publication does not pertain to healthcare context (e.g., animal research, environmental or business studies) |
|  | Publication does not provide elaborate description of definition, requirements, operationalisation or challenges of a social licence |
|  | Publication does not pertain to research |
|  | Publication not written in English |
|  | No full-text available |
