# Supplementary material for: The social licence for data-intensive health research: towards co-creation, public value and trust
Source: BMC Med Ethics. 2021 Aug 10;22:110. doi: 10.1186/s12910-021-00677-5 (PMC8353823; doi:10.1186/s12910-021-00677-5)
Supplement: Supplementary file 3 — Additional file 3:Figure: Figure containing flow diagram of the selection of publications. [file 12910_2021_677_MOESM3_ESM.docx]

Additional file 3. Figure containing flow diagram of the selection of publications.

Records excluded after full-text screening
(n = 14)

Additional records identified through other sources
(n = 1)

Total records included for analysis (n = 9)

Records included for analysis (n = 8)

Full-text articles assessed for eligibility
(n = 22)

Records excluded after Title/Abstract screening (n = 131)
(n = 8)

Total unique records
(n = 153)

Scopus
(n = 111)

Duplicates excluded
(n = 67)

Embase
(n = 49)

PubMed
(n = 60)
